# Development and validation of a short-term breast health measure as a supplement to screening mammography

**DOI:** 10.1186/s40364-022-00420-1

**Published:** 2022-10-25

**Authors:** Anna Daily, Prashanth Ravishankar, Wanyi Wang, Ryan Krone, Steve Harms, V. Suzanne Klimberg

**Affiliations:** 1 Namida Lab Inc., Fayetteville, AR USA; 2 Elite Research, LLC, Irving, TX USA; 3 The Breast Center-Medical Associates of Northwest Arkansas, Fayetteville, AR USA; 4 Department of Surgery, University of Texas Medical Branch, Galveston, TX USA; 5 Breast Surgical Oncology, The University of Texas MD Anderson Cancer Center, Houston, TX USA

**Keywords:** Breast cancer, Cancer biomarkers, Biomarker validation, Tear fluids, ELISA, Receiver operator characteristic curves

## Abstract

**Background:**

There is a growing body of evidence to support tears as a non-traditional biological fluid in clinical laboratory testing. In addition to the simplicity of tear fluid processing, the ability to access key cancer biomarkers in high concentrations quickly and inexpensively is significantly enhanced. Tear fluid is a dynamic environment rich in both proteomic and genomic information, making it an ideal medium for exploring the potential for biological testing modalities.

**Methods:**

All protocols involving human subjects were reviewed and approved by the University of Arkansas IRB committee (13-11-289) prior to sample collection. Study enrollment was open to women ages 18 and over from October 30, 2017-June 19, 2019 at The Breast Center, Fayetteville, AR and Bentonville, AR. Convenience sampling was used and samples were age/sex matched, with enrollment open to individuals at any point of the breast health continuum of care. Tear samples were collected using the Schirmer strip method from 847 women. Concentration of selected tear proteins were evaluated using standard sandwich ELISA techniques and the resulting data, combined with demographic and clinical covariates, was analyzed using logistic regression analysis to build a model for classification of samples.

**Results:**

Logistic regression analysis produced three models, which were then evaluated on cases and controls at two diagnostic thresholds and resulted in sensitivity ranging from 52 to 90% and specificity from 31 to 79%. Sensitivity and specificity variation is dependent on the model being evaluated as well as the selected diagnostic threshold providing avenues for assay optimization.

**Conclusions and relevance:**

The work presented here builds on previous studies focused on biomarker identification in tear samples. Here we show successful early classification of samples using two proteins and minimal clinical covariates.

**Supplementary Information:**

The online version contains supplementary material available at 10.1186/s40364-022-00420-1.

## Background

Breast cancer is the most diagnosed cancer in women and accounts for up to 31% of all cancer types [1, 2]. It is estimated that 43,250 deaths would be associated with breast cancer in 2022 in the US [3]. The most effective way to reduce mortality associated with breast cancer is to increase early detection and therefore intervention leading to proper treatment [4–6].

The early 1980’s saw a major advancement in breast cancer detection due to the implementation of screening mammography; prior to which, most breast cancers were detected by a palpable mass or breast abnormality found by a physician or patient [7]. With screening mammogram, image-based detection jumped from 4% in 1977 to 41% in 1988 with a mortality reduction of 20% [8, 9]. As the technology advanced from film to digital and now to 3D, the detection rate is steady around 60% [10]. Surprisingly, with all of the advancement and promotion of screening mammography, the rate of so-called interval cancers has remained unchanged with a significant increase in the rate of false-positives requiring follow-up [11].

Mammography correctly detects roughly 87% of patients with breast cancer (76% specificity and 87% sensitivity), and this sensitivity rises with age and in women with fatty breast tissue [12–14]. According to a 2017 study, 63.4% of diagnosed breast cancers were stage 0 or stage 1, and 69.6% of invasive cancers were lymph node negative, based on over 400,000 diagnostic imaging breast examinations performed at 92 different radiology centers [15].

Despite the obvious necessity for screening mammography in the reduction of mortality due to breast cancer, adherence to this modality, based on age, is anywhere from 31 to 70% of women [16]. Typically, compliance with screening increases as women age which also correlate with an increased risk of breast cancer. However, the rate of breast cancer diagnosis in women under 50 is steadily increasing and those diagnoses are typically more aggressive and have higher mortality rates [17]. Unfortunately, the same population has the lowest compliance rates with screening mammography. A recent report by radiologists revealed that the mortality rate of breast cancer in women aged 20–39 has stopped declining since 2010 and increased by 0.5% per year [18]. Screening recommendations in this age range (< 50) vary and this period in women’s lives is generally when the demand of career and family responsibilities are highest and available time for personal care is lowest. According to a 2020 study, screening mammography in women aged 40 to 49 years reduced mortality by approximately 25% in the first 10 years when compared to waiting until 50 years or older to begin screening [19].

Another factor effecting the accuracy of mammography is breast density. High mammographic density decreases the diagnostic accuracy of screening mammography by masking tumors and is a risk factor for breast cancer on its own. Breast density is connected with young age, pregnancy, lactation, and hormonal treatment [20–22]. In the BI-RADS lexicon, there are four descriptors for breast density, (1) fatty, (2) scattered areas of fibroglandular density, (3) heterogeneously dense, which can obscure small masses, and (4) extremely dense, which reduces mammographic sensitivity [23].

We use tears as our biofluid source for analyzing the biomarkers for breast cancer. It has been documented that systemic effects exert influence on the ocular environment [24]. Tear fluid is a dynamic environment rich in both proteomic and genomic information [25–27]. Studies show that protein patterns in tears have the potential to generate biomarkers for disease state determination and could also provide new sources for treatment options and monitoring [28–30]. Most promising new discoveries in protein biomarkers focus on low molecular weight proteins which in many cases are undetectable without significant pre-processing of the samples [31]. There is a growing body of evidence to support tears as a non-traditional biological fluid [32–34]. In addition to the simplicity of tear fluid processing, the ability to access key cancer biomarkers in high concentrations quickly and inexpensively is significantly enhanced.

We had previously conducted a biomarker discovery study where we reported the 3 proteins namely S100A8, S100A9, and Galectin-3 binding proteins as potential biomarker candidates [35]. Each of these proteins play a major role in the development of breast cancer. S100 proteins’ role in cancer is well documented [36]. Previous research groups have reported that S100A8 and S100A9 were elevated in serum and tissue of breast cancer patients [37–40]. Additionally, the increased expression of these two S100 proteins have been associated with non-functional BRAC1 which play a role in metastasis by binding to RAGE receptors [41]. Galectin-3 binding proteins have been shown to be a potential binding site for proteins involved in metastasis and the protein’s elevated levels are associated with shorter survival in patients with breast carcinoma [42, 43]. In this study we validate and build on our previous work [35], using a larger patient population and analysis of clinical covariates. By compiling the scores of the large case-control study, we provide the foundation for a “pre-screen” for women with low to average lifetime risk of breast cancer without a palpable mass or area of breast concern as supplement to screening mammography.

## Methods

### Study population

All protocols involving human subjects were reviewed and approved by the University of Arkansas IRB committee (13-11-289) prior to sample collection. The sampling technique used was a purposive, non-random sampling strategy to recruit women with the requisite inclusion criteria. Tear fluid samples were collected from study participants recruited at The Breast Center, Fayetteville, AR and Bentonville, AR. Written informed consent was obtained from all participants prior to sample collection. Patients were given the opportunity to enroll if they were being seen for standard yearly screening, imaging to evaluate an area of breast concern, biopsy, and recently diagnosed with breast cancer being evaluated for pre-surgical MRI evaluation. Imaging results, from the procedure at sample collection, as well as any follow-up imaging was obtained through The Breast Center to assist with sample classification. Details for sample classifications are displayed in Table 1.

**Table 1:** Sample classifications and qualifications

Sample Class	BiRADS	Imaging procedure at sample collection	Outcome
Normal	1,2	Screening MGM- no documented area of concern	Classified as normal and a recommendation to continue normal screening
Call-back	1,2	Screening MGM, or diagnostic - may have documented area of concern	Call-back for further imaging that resulted in a “normal” classification and a return to normal screening.
Category 3	3	Screening or diagnostic - may have documented area of concern	Call-back for further imaging that resulted in a recommendation for additional follow-up.
Benign	4a, 4b, 4c	Screening, Diagnostic, Biopsy - may have documented area of concern	Biopsy with final diagnosis as Benign
Breast Cancer	4a, 4b, 4c, 5	Screening, Diagnostic, Biopsy, MRI - may have documented area of concern	Biopsy confirmed Breast Cancer diagnosis

### Sample preparation and ELISA

Tear fluid samples were collected and evaluated for the expression level of S100A8, S100A9, and Galectin-3-Binding Protein (LG3BP) using ELISA (DuoSets ELISA kits, R&D Systems (Minneapolis, MN, USA) based on previously reported protocols [35].

### Statistical analysis

R statistical software was used to apply logistic regression to protein concentrations determined by ELISA for comparison. Forward stepwise logistic regression was used to determine diagnostic parameters for the optimal combinations of proteins of interest and subject demographic characteristics. This was done to reduce the number of predictors in the model to a more parsimonious set. The algorithm derived from the logistic regression model was then used to calculate predicted probability scores for each subject. Receiver operator characteristic (ROC) curves were generated using these predicted probability scores against the breast cancer dependent variables and the area under the curve (AUC) was computed accordingly. An AUC of 0.7 or greater was set as the standard of acceptance for a panel of proteins and characteristics. Sensitivity (true positive rate) and specificity (true negative rate) scores were also calculated to assess the accuracy of the test. The data preparation and analysis were conducted in SPSS version 25 [44]. Crosstabulations using chi-square (Pearson’s χ^2^) tests were performed to examine the relationships between each diagnosis (sensitivity/specificity/accuracy) and three models in each scenario, and a *p*-value < 0.05 was considered statistically significant.

## Results

### Sample population

Logistic regression models were built using a cohort dataset of 391 samples (Cohort 1) collected from a single site from October 2017 through December 2018. As shown in Table 2, age range of the entire study population was 22–84 years of age, with an average of 55.81 ± 12.22 yrs. of age. At the conclusion of enrollment, the study population consisted of 87 confirmed breast cancer cases with a subtype distribution of IDC (55%), DCIS (23%), ILC (5%), multiple diagnosis (5%) (i.e. DCIS/IDC, ILC/IDC), remaining diagnosis (13%) comprising, infiltrating mammary carcinoma; infiltrating mammary duct carcinoma; invasive ductal carcinoma; metastatic mammary carcinoma; papillary carcinoma; and invasive cribriform carcinoma. Controls were divided into two groups, Normal, taken from subjects who were not called-back for additional imaging after a screening mammogram; and Call-back, taken from subjects who were recalled but the recall did not lead to biopsy, and they were cleared to return to standard yearly screening.

**Table 2:** Demographics of patient database

Category	Cohort 1 (*n* = 391)	Cohort 2 (*n* = 456)	Combined (*n* = 847)
Age, *y*, median (range)	57 (22–87)	53 (21–87)	55 (21–87)
Normal			
*Normal screening mammogram*	223	145	368
Call-Back			
*Normal Diagnostic Mammogram*	81	83	164
Cases			
*Biopsy Confirmed Breast Cancer*	87	21	108
IDC	48	14	62
ILC	4	3	7
DCIS	20	2	22
Multiple	4	0	4
Other	11	2	13
Grade			
Low	18	2	20
Intermediate	41	11	52
High	26	7	33
Unknown	2	1	3
Tumor Size (cm)			
< 1	28	3	31
1–2	27	11	38
> 2	29	7	36
Multiple	9	2	11
Primary tumor with positive node	15	2	17
Unknown	3		3
Receptor Status			
ER−/PR-	1	1	2
ER−/PR+	1	0	1
ER+/PR+	16	2	18
ER+/PR-	3	0	3
ER−/PR−/HER2+	3	0	3
ER+/PR+/HER2-	47	13	60
ER+/PR−/HER2-	4	1	5
ER+/PR+/HER2+	8	2	10
Triple Negative	4	2	6
Benign		121	
Category 3		86	

Another cohort dataset of 456 samples (Cohort 2) with an average 53.48 ± 12.14 yrs. of age were collected from the same single site from December 2018 through June 2019. The data set consisted of an additional 21 breast cancer samples with a subtype distribution of IDC (67%), ILC (14%), DCIS (10%), with the remaining falling into the “other” category (10%). Additionally, this dataset contained 121 biopsy confirmed benign samples as well as 86 samples assigned BiRADS 3 with recommendation for short-term follow-up. A summary of sample characteristics for the Cohort 1 and 2 can be found in Table 2.

### Logistic regression

Stepwise forward logistic regression analysis was used to identify distinguishing covariate combinations. Due to the non-normal distribution of the three protein analytes (S100A8, S100A9, LG3BP), concentrations were transformed to log values for the analysis. In instances where the average protein values were 0 pg/ml, effectively below the lower limit of detection, the value was recoded to 1 pg/ml enabling the log(protein) to be taken generating a 0 instead of missing.

After assessment for collinearity of the 28 covariates, 11 potential covariates remained for evaluation- three proteins (S100A8, S100A9, LG3BP), age, BMI, HRT, family history of cancer, family history of breast cancer, personal history of cancer, personal history of breast cancer, and breast density. Logistic regressions were conducted on the data set in three conditions; Model 1- Normal vs. Breast Cancer (1); Model 2- Call-back vs. Breast Cancer (2); Model 3- Normal & Call-back vs. Breast Cancer (3), and the Forward Logistic Regression Algorithm formulas are provided below with Y representing the scores for each model,

1 $$Model\ 1:\textrm{Y}=-6.64+{1.04}^{\ast}\left(\textrm{logS}100\textrm{A}8\right)+{0.811}^{\ast}\left(\textrm{logS}100\textrm{A}9\right)+{0.62}^{\ast}\left(\textrm{breast}\ \textrm{density}\right)$$

2 $$Model\ 2:\textrm{Y}=-6.988+{0.614}^{\ast}\left(\textrm{logS}100\textrm{A}8\right)+{1.081}^{\ast}\left(\textrm{logS}100\textrm{A}9\right)+{0.034}^{\ast}\left(\textrm{age}\right)$$

3 $$Model\ 3:\textrm{Y}=-7.244+{0.955}^{\ast}\left(\textrm{logS}100\textrm{A}8\right)+{1.047}^{\ast}\left(\textrm{logS}100\textrm{A}9\right)$$

### Validation of the models on cohort 1

**Fig. 1:**
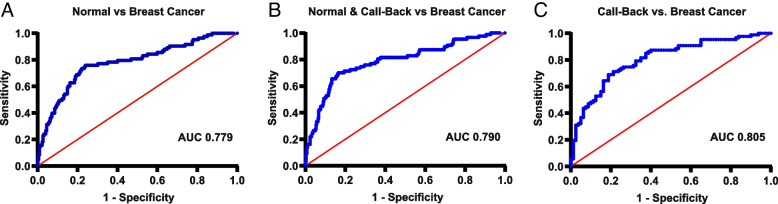
**A** Receiver Operating Characteristic (ROC) Curves comparing Normal versus Breast Cancer with an AUC of 0.779; **B** Call-back versus Breast Cancer with an AUC of 0.805; **C** Normal & Call-back versus Breast Cancer with an AUC of 0.790

Receiver operator characteristic curves (ROC) were generated for each model on the cohort 1 dataset and used to select various diagnostic thresholds for analysis (Fig. 1). For each model, two diagnostic thresholds were selected; Scenario 1 utilized the Y (score) where the sum of sensitivity and specificity was maximized. For scenario 2, a Y (score) was selected with preference given to 90% sensitivity to evaluate the potential reduction of false negatives.

For the scenario 1 in the cohort 1 dataset, analysis of Normal vs. Breast Cancer samples produced model 1 which incorporated S100A8, S100A9, and breast density as the predictors. The overall AUC was 0.779 with a maximized sensitivity of 76% and a specificity of 76% when the cut-off point was set as Y > − 0.8565. These results along with the positive coefficient of the three predictors indicate that S100A8, S100A9, and breast density were significantly associated with a positive breast cancer diagnosis. The AUC score of 0.779 further suggests an acceptable factor combination to predict breast cancer.

Analysis of Call-back vs. Breast Cancer samples produced model 2 which incorporated S100A8, S100A9, and age as the predictors. An AUC of 0.805 revealed that this algorithm is a good indicator of the breast cancer diagnosis with a maximized sensitivity of 70% and a specificity of 81% when the cut-off point was set as Y > 0.2018.

Analysis of Normal & Call-back vs. Breast Cancer samples produced a model 3 with S100A8 and S100A9 as the predictors. An AUC of 0.790 suggests a fair to good indicator of the breast cancer diagnosis. When the cut-off value Y was set above − 0.9487, sensitivity and specificity were maximized to 70 and 84% respectively.

### Evaluation of the models on the combined dataset

**Fig. 2:**
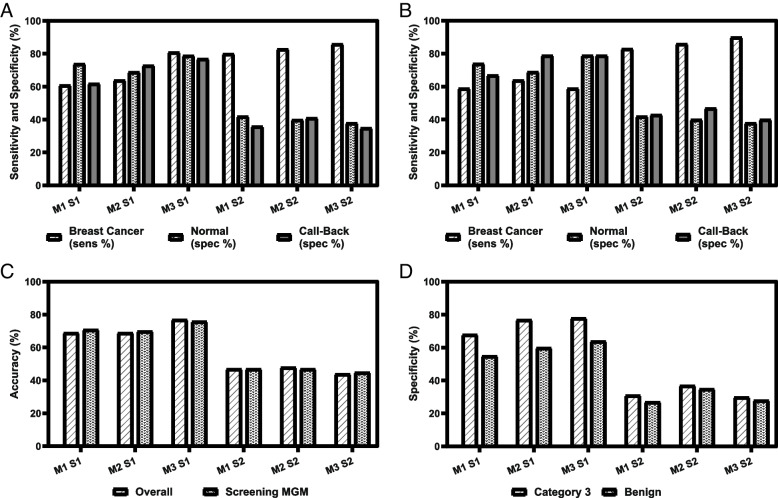
**A** Comparisons of performance threshold values from the three models (M) produced by logistic regression analysis at two different scenarios (S). **B** Selected samples where the patient entered the continuum of care at the screening mammogram. **C** Accuracy rates for all screening modalities versus accuracy rates for individuals who entered the continuum of care at screening mammogram only. **D** Finally, shown is specificity for biopsy confirmed benign samples as well as samples assigned a BiRADs 3 score

Diagnostic parameters (sensitivity and specificity) for scenarios 1 and 2 for models 1, 2, & 3 were evaluated for the entire 847 sample data set to find the most effective model out of all three proposed models. In each case, samples were considered positive if the Y-score was greater than the cut-off and below which they were considered negative. Scenario 1 with Model 1 (M1 S1), a Y-score of − 0.8565 resulted in a sensitivity of 61%, specificities of 74 and 62% for Normal & Call-back respectively, and an overall accuracy of 69% (Fig. 2A and C). For model 2 scenario 1 (M2 S1), a Y-score of 0.2018 was used, producing a sensitivity of 64%, specificities of 69 and 73% for Normal & Call-back respectively, and an overall accuracy of 69%. Finally, for model 3 scenario 1 (M3 S1) with a Y-score of − 0.9487 resulted in a sensitivity of 81% and specificities of 79 and 77% for Normal & Call-back respectively, and an overall accuracy of 77%. A crosstabulation using a Pearson χ^2^ test was conducted and found that there was no statistically significant relationship between diagnosis and M1/M2/M3 for sensitivity and accuracy. However, there was a statistically significant relationship between specificity of normal control and three models in scenario 1 (*p* = 0.008). Model 3 resulted in highest specificity in the prediction of normal population (79%) whereas model 2 had lowest specificity in the prediction of normal population (69%). The significant relationship was also found between the three models and the specificity of call-back controls in scenario 1 (*p* = 0.007). A greater specificity of call-back controls was predicted in the model 3 (77%) as compared to the model 1 (62%) (See Fig. 2A).

A Y-score of − 1.8236 was used for Scenario 2 for model 1 (M1 S2), and this resulted in a sensitivity of 80% and specificities of 42 and 36% for Normal & Call-back respectively, and an overall accuracy of 47%. Model 2 scenario 2 (M2 S2) with Y-score of − 0.828 produced a sensitivity of 83% and specificities of 40 and 41% for Normal & Call-back respectively, and an overall accuracy of 48%. Model 3 scenario 2 (M3 S2) utilized a Y-score of − 2.3226 resulting in a sensitivity of 86% and specificities of 38 and 35% respectively for Normal & Call-back respectively, and an overall accuracy of 44%. A Pearson χ^2^ test revealed no statistically significant relationship between diagnosis and M1/M2/M3 for sensitivity, specificity, and accuracy.

All three models and scenarios were then applied to our entire dataset where the participant had entered the breast health continuum of care at screening mammogram to evaluate how the models would perform in a pre-screening application (Fig. 2B and C). For M1 S1 sensitivity was 59% and specificities were 74 and 67% for Normal & Call-back respectively, and an overall accuracy of 71%. M2 S1 produced a sensitivity of 64% and specificities of 69 and 79% for Normal & Call-back respectively, and an overall accuracy of 70%. M3 S1 produced a sensitivity of 59% and specificities of 79 and 79% for Normal & Call-back respectively, and an overall accuracy of 76%. There was no statistically significant relationship between diagnosis and M1/M2/M3 for sensitivity and accuracy. However, there was a statistically significant relationship for specificity in the normal controls (*p* = 0.008) and a trend was observed in call-back controls (*p* = 0.093). A greater specificity of Normal population was predicted in the model 3 (79%) as compared to the model 2 (69%) (See Fig. 2B).

For the second scenario, M1 S2 resulted in a sensitivity of 83% and specificities of 42 and 43% for Normal & Call-back respectively, and an overall accuracy of 47%. M2 S2 produced a sensitivity of 86% with specificities of 40 and 47% for Normal & Call-back respectively, and an overall accuracy of 47%. M3 S2 produced a sensitivity of 90% and specificities of 36 and 40% for Normal & Call-back respectively, and an overall accuracy of 45%. A Pearson χ^2^ test revealed no statistically significant relationship between diagnosis and M1/M2/M3 for sensitivity, specificity, and accuracy.

The final set of samples in the population pool consisted of category 3 and benign to evaluate how the models would perform in a diagnostic application (Fig. 2D). M1 S1 resulted in a specificity of 68 and 55% for category 3 and benign respectively. M2 S1 performed better with a 77% specificity for category 3 and 60% for benign; and M3 S1 produced a specificity of 78% for category 3 and 64% for benign. For the second scenario, M1 S2 resulted in a specificity of 31% for category 3 and 27% for benign. M2 S2 resulted in 37% specificity for category 3 and 35% for benign. Finally, M3 S2 showed a specificity of 30% for category 3 and 28% for benign. There was no statistically significant relationship between category 3, benign, and the three models for specificity in both the scenarios.

## Discussion

Here we provide an analysis of the potential capability of tear proteins to be used in the classification of control and breast cancer samples. In this study, we analyze 11 potential clinical covariates, by logistic regression to develop a diagnostic algorithm for sample classification. Methods for the selection of protein biomarkers included in the analysis, S100A8, S100A9, and LG3BP were described previously [35]. Breast cancer samples were compared to two different groups- Normal & Call-back, as the final diagnosis for women in both groups was “normal” for that year, however subjects imaging path differed (Table 1). Individuals in the call-back group experienced an additional imaging step consisting of diagnostic mammogram and in some cases, a diagnostic ultrasound. Women in this group were not recommended to have an additional confirmatory imaging after the diagnostic and were returned to a yearly screening cycle. The call-back group was analyzed separate from normal because reducing false-positive call-backs from imaging is a high priority in breast imaging.

The clinical covariates included in the models were the main differentiating factor as all three utilized S100A8 and S100A9. Model 1 incorporated breast density, model 2 incorporated age, while model 3 only utilized the two protein concentrations. Comparison of the model performance against one another is essential as each model was developed using specific portions of the dataset. Consideration of the covariates dictate where a model could be used in the breast health continuum of care. For example, utilization of model 1 as pre-screening tool would not be feasible as model 1 requires information about tissue density category. Establishment of tissue density is done after imaging and may also change through the course of a woman’s lifetime. Additionally, since model 1 had the lowest performance after initial assessment, models 2 and 3 were the focus of further application. The performance of all three models on the entire data set were evaluated using Pearson’s χ^2^ test and it revealed no statistically significant relationship between diagnosis and M1/M2/M3 for sensitivity, specificity, and accuracy providing evidence that any of the three models could be selected moving forward.

The lower specificity values ranging between 35 and 45% are associated with scenario two for each model. In scenario two, the clinical threshold was selected preferential to sensitivity. Because of the dynamic relationship between sensitivity and specificity, when preference is given to one value the other value often decreases. Scenario 1 in each model tests the y-value where both the sensitivity and specificity are at the highest. For Model 1 the y-value was set at − 0.8565 which provided a sensitivity of 75.9% and specificity of 76.1%. When the y-value was preferentially adjusted for higher sensitivity to − 1.8236 the predicted sensitivity is now 90% and specificity is 34%. Desired clinical outcomes play a significant role in the selection of the diagnostic threshold. In thinking about the implications of false negatives and false positives, the first preference is to get both numbers as low as possible however if the false negative rate is too high then the threshold is adjusted.

The diversity of diagnosis included in the breast cancer samples allowed for investigation into the performance of the models by cancer subtypes, cancer grade, and tumor size including a small portion of subjects with node metastasis and the relevant sensitivities are reported in the supplemental section. Diagnostic thresholds M2 S2 and M3 S2 were used to evaluate the performance (Supplemental Fig. 1). While tumor size is not an indication of disease severity, it is of interest when considering utility of a biological test prior to screening mammography. Ideally, these models should perform best in a normal breast prior to palpability of a lump or identification of concern. Both models performed best in smaller tumors and lowest performance was in subjects with multiple breast tumors identified or where metastasis to a node had occurred. We observed a trend when assessing sensitivity of the models by grade where low and intermediate grades of cancer had better sensitivities. The performance of the models for lower grade cancer is not surprising given the roles of S100A8 and S100A9 in recruitment of immune cells essentially prepping the tissue for tumor formation [45–47].

Utilization of screening mammography among insured women in the US hovers around 60% with the lowest participation of 35% in women under 45 years of age [48]. While the detection rate in women under 40 is only 6.5%, these diagnoses are often more aggressive [49]. Screening can only be effective if utilized. It is possible that a simple test offered prior to screening mammography for low to average risk women could increase participation in yearly screening mammography. While there is more development work to be done, given the elegant simplicity of tear sample collection, this could be an interesting medium to explore for a “pre-screening” application.

Limitations of the study include, only one clinical location which limited geographic, racial, ethnic, and economic distribution of subjects. In addition, evaluation of S100A8 and S100A9 has been limited to only breast cancer. Future studies will incorporate additional cancer subtypes. Additionally, most recent developments have focused on employing machine learning tools to develop better diagnostic algorithms and along the same lines, our future work will also focus on developing models with increased specificity by studying more clinical covariates.

## Conclusions

In this study, we used tear fluids to determine that cancer biomarkers’ protein concentrations and developed a model that is significantly associated with a positive breast cancer diagnosis. We analyzed the protein concentration from 847 individually collected tears samples using logistic regression to develop and validate three models for the identification of positive breast cancer samples with a sensitivity as high as 90%. Our analysis suggests that models developed using tear fluid have clinical validity and could be used in further development of a biological assay to supplement screening mammography for screening adverse individuals or in areas where access to screening mammography is limited.

## Supplementary Information

**Additional file 1: Fig. 1.** Sensitivity of Model 2 and Model 3 according to A. breast cancer subtype, B. grade, and C. tumor size.

## Data Availability

All data that support the findings of this study are available from the corresponding authors on reasonable request.

## Abbreviations

BI-RADS: Breast imaging reporting and data system
ROC: Receiver operating characteristics
AUC: Area under the curve
LG3BP: Galectin-3-binding protein
IDC: Intraductal carcinoma
DCIS: Ductal carcinoma in situ
