# Supplementary figures and images for: Development and validation of a short-term breast health measure as a supplement to screening mammography
Source: Biomark Res. 2022 Oct 25;10:76. doi: 10.1186/s40364-022-00420-1 (PMC9594920; doi:10.1186/s40364-022-00420-1)

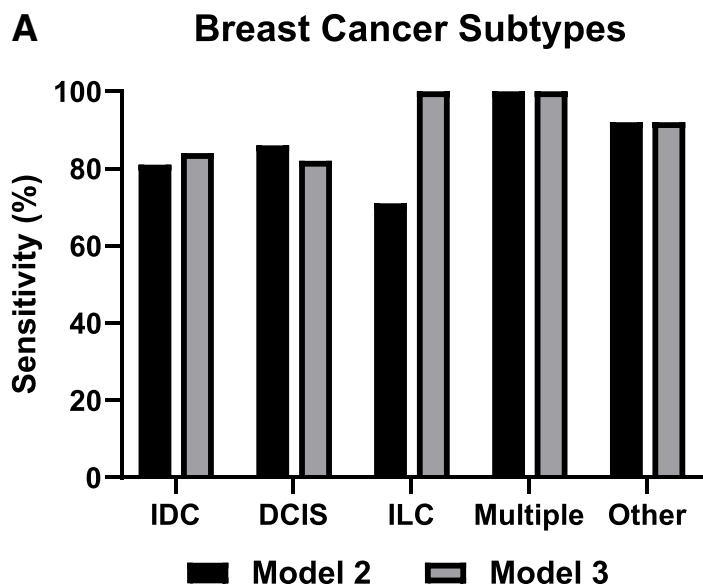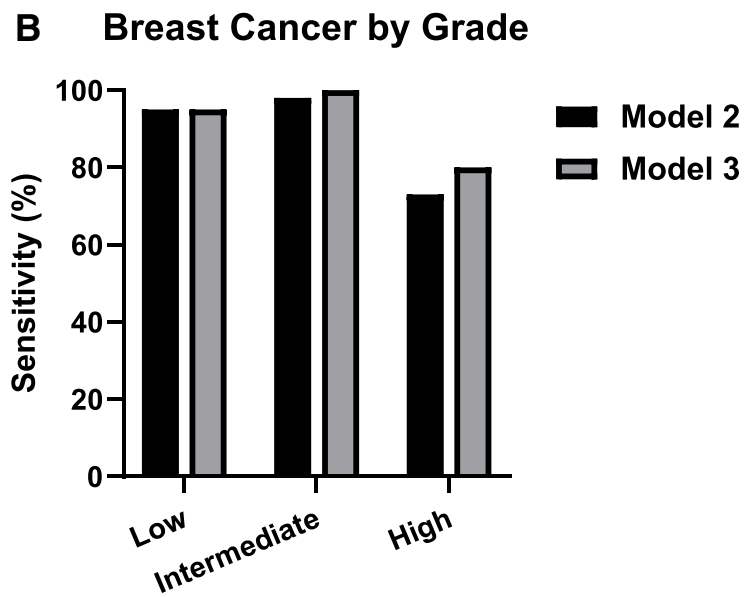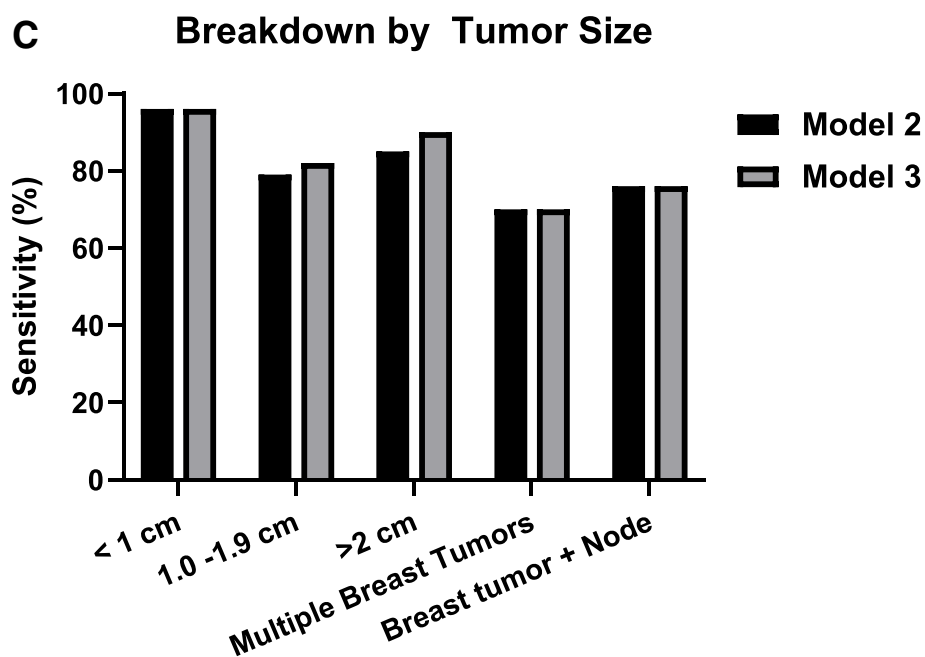

Supplement: Supplementary file 1 — Additional file 1: Fig. 1. Sensitivity of Model 2 and Model 3 according to A. breast cancer subtype, B. grade, and C. tumor size. [file 40364_2022_420_MOESM1_ESM.pdf]
